# Basic inequality on a *b*-metric space and its applications

**DOI:** 10.1186/s13660-017-1528-3

**Published:** 2017-10-11

**Authors:** Tomonari Suzuki

**Affiliations:** Department of Basic Sciences, Faculty of Engineering, Kyushu Institute of Technology, Tobata, Kitakyushu, 804-8550 Japan

**Keywords:** 54E99, 54H25, *b*-metric space, Cauchy sequence, fixed point theorem

## Abstract

We first prove one of the most basic inequalities on a *b*-metric space. And then we prove some fixed point theorems. We also consider two similar conditions; one implies the Cauchyness on sequences but the other does not.

## Introduction

In 1998, Czerwik introduced the following interesting concept.

### Definition 1

(Czerwik [1])

Let *X* be a set and let *d* be a function from $X \times X$ into $[0, \infty)$. Then $(X,d)$ is said to be a *b-metric space* if the following hold:

1. $d(x,y) = 0 \Leftrightarrow x = y$;
2. $d(x,y) = d(y,x)$ (symmetry);
3. There exists $K \geq1$ satisfying $d(x,z) \leq K ( d(x,y) + d(y,z) )$ for any $x, y, z \in X$ (*K*-relaxed triangle inequality).

We note that in the case where $K = 1$, every *b*-metric space is obviously a metric space. So this concept is a weaker concept than that of a metric space. Conditions (b1) and (b2) also appear in the definition of metric space. So (b3) is a feature of this concept. Therefore it is important to study how to use (b3) effectively.

### Lemma 2

*Let* $(X,d)$ *be a* *b*-*metric space*. *For* $n \in \mathbb {N}$ *and* $(x_{0},\ldots,x_{n}) \in X^{n+1}$,

1 $$ d(x_{0},x_{n}) \leq\sum _{j=0}^{n-2} K^{j+1} d(x_{j}, x_{j+1}) + K^{n-1} d(x_{n-1}, x_{n}) $$

*holds*.

### Proof

Obvious. □

Considering the rearrangement inequality, we could tell that (1) is effective in the case where $d(x_{j+1}, x_{j+2})$ is much smaller than $d(x_{j}, x_{j+1})$. Indeed, using (1), the following lemma was proved.

### Lemma 3

(Lemma 3.1 in [2])

*Let* $(X, d)$ *be a* *b*-*metric space and let* $\{ x_{n} \}$ *be a sequence in X*. *Assume that there exists* $r \in[0,1/K)$ *satisfying* $d(x_{n+1}, x_{n+2}) \leq r d(x_{n}, x_{n+1})$ *for any* $n \in \mathbb {N}$. *Then* $\{ x_{n} \}$ *is Cauchy*.

In the case where $d(x_{j+1}, x_{j+2})$ is not much smaller than $d(x_{j}, x_{j+1})$, how can we use (b3) effectively?

Motivated by this question, in this paper, we first prove some inequality. Using this inequality, we improve Lemma 3. In order to understand deeply the mathematical structure of a *b*-metric space, we give a condition, which does not imply the Cauchyness on sequences. Finally, we improve some Nadler-type fixed point theorems.

## Preliminaries

Throughout this paper we denote by $\mathbb {N}$ the set of all positive integers and by $\mathbb {R}$ the set of all real numbers. For an arbitrary set *A*, we also denote by #*A* the cardinal number of *A*. For a real number *t*, we denote by $[t]$ the maximum integer not exceeding *t*.

In this section, we give some preliminaries.

### Definition 4

Let $(X,d)$ be a *b*-metric space. Let $\{ x_{n} \}$ be a sequence in *X* and let *A* be a subset of *X*.

- $\{ x_{n} \}$ is said to *converge* to *x* if $\lim_{n} d(x, x_{n}) = 0$ holds.
- $\{ x_{n} \}$ is said to be *Cauchy* if $\lim_{n} \sup\{ d(x_{n}, x_{m}) : m > n \} = 0$ holds.
- *X* is said to be *complete* if every Cauchy sequence converges.
- *A* is said to be *closed* if for any convergent sequence in *A*, its limit belongs to *A*.
- *A* is said to be *bounded* if $\sup\{ d(x,y) : x, y \in A \} < \infty$ holds.

### Remark

While not every *ν*-generalized metric space is metrizable [3–5], every *b*-metric space is metrizable. So we note that there is no room for ambiguity in Definition 4.

Let $(X,d)$ be a *b*-metric space and let $\operatorname {CB}(X)$ be the set of all nonempty, bounded and closed subsets of *X*. For $x \in X$ and any subset *A* of *X*, we define $d(x,A) = \inf\{ d(x,y) : y \in A \}$. Then the *Hausdorff metric* *H* with respect to *d* is defined by

$$ H(A,B) = \max \bigl\{ \sup\bigl\{ d(u,B) : u \in A \bigr\} , \sup\bigl\{ d(v,A) : v \in B \bigr\} \bigr\} $$

for all $A,B \in \operatorname {CB}(X)$.

## Basic inequality

In this section, we prove one of the most basic inequalities on a *b*-metric space.

### Lemma 5

*Let* $(X,d)$ *be a* *b*-*metric space*. *Define a function* *f* *from* $\mathbb {N}$ *into* $\mathbb {N}\cup\{ 0 \}$ *by*

2 $$ f(n) = - [- \log_{2} n] . $$

*For* $n \in \mathbb {N}$ *and* $(x_{0},\ldots,x_{n}) \in X^{n+1}$,

3 $$ d(x_{0},x_{n}) \leq K^{f(n)} \sum _{j=0}^{n-1} d(x_{j}, x_{j+1}) $$

*holds*.

### Remark

*f* is as follows:

$$\begin{aligned} & f(1)=0, \quad\quad f(2) = 1, \quad\quad f(3) = f(4) = 2, \\ & f(5) = \cdots= f(8) = 3 , \quad\quad f(9) = \cdots= f(16) = 4 ,\quad\quad \ldots. \end{aligned}$$

### Proof

We first note that $2^{f(n)-1} < n \leq2^{f(n)} $ holds for any $n \in \mathbb {N}$. It is obvious that (3) holds for $n = 1$. We assume that (3) holds for *n* with $n \leq2^{k}$ for some $k \in \mathbb {N}\cup\{ 0 \}$. Fix $n \in \mathbb {N}$ with $2^{k} < n \leq2^{k+1}$. Then we note $f(n) = k+1$, $f(2^{k}) = k$ and $f(n - 2^{k}) \leq k$. We have

$$\begin{aligned} d(x_{0},x_{n}) &\leq K d(x_{0},x_{2^{k}}) + K d(x_{2^{k}}, x_{n}) \\ &\leq K K^{f(2^{k})} \sum_{j=0}^{2^{k}-1} d(x_{j},x_{j+1}) + K K^{f(n-2^{k})} \sum _{j=2^{k}}^{n-1} d(x_{j},x_{j+1}) \\ &\leq K^{k+1} \sum_{j=0}^{n-1} d(x_{j},x_{j+1}) \\ &= K^{f(n)} \sum_{j=0}^{n-1} d(x_{j},x_{j+1}) . \end{aligned}$$

Thus (3) holds for *n*. By induction, we obtain the desired result. □

Using Lemma 5, we give a sufficient condition for the Cauchyness on sequences. The following lemma can be very useful when we prove existence theorems in complete *b*-metric spaces. In order to demonstrate that, in Section 6, we improve some fixed point theorems. See [6, 7] and others.

### Lemma 6

*Let* $(X, d)$ *be a* *b*-*metric space and let* $\{ x_{n} \}$ *be a sequence in* *X*. *Assume that there exists* $r \in[0,1)$ *satisfying*

4 $$ d(x_{n+1}, x_{n+2}) \leq r d(x_{n}, x_{n+1}) $$

*for any* $n \in \mathbb {N}$. *Then* $\{ x_{n} \}$ *is Cauchy*.

### Remark

Compare Lemma 6 with Lemma 3.

### Proof

In the case where $r = 0$, the conclusion obviously holds. So we assume $r > 0$. We choose $\ell\in \mathbb {N}$ satisfying

$$ K r^{2^{\ell}} < 1 . $$

Define a function *f* by (2). For $m, n \in \mathbb {N}$ with $n < m \leq n+2^{\ell}$, we have by Lemma 5

$$\begin{aligned} d(x_{n}, x_{m}) &\leq K^{f(m-n)} \sum _{j=n}^{m-1} d(x_{j}, x_{j+1}) \\ &\leq K^{\ell} \sum_{j=n}^{m-1} r^{j-1} d(x_{1}, x_{2}) \\ &\leq K^{\ell} \sum_{j=n}^{\infty}r^{j-1} d(x_{1}, x_{2}) \\ &\leq K^{\ell} r^{n} C , \end{aligned}$$

where we put $C = d(x_{1}, x_{2}) / ( r (1-r) )$. For $m, n \in \mathbb {N}$ with $n+2^{\ell}< m$, putting $\mu= [(m-n)/2^{\ell}]$, we have by (1)

$$\begin{aligned} d(x_{n}, x_{m}) &\leq\sum_{i=0}^{\mu-1} K^{i+1} d(x_{n+i 2^{\ell}}, x_{n+(i+1) 2^{\ell}}) + K^{\mu} d(x_{n+\mu 2^{\ell}}, x_{m}) \\ &\leq\sum_{i=0}^{\mu-1} K^{i+\ell+1} r^{n+i 2^{\ell}} C + K^{\mu+\ell} r^{n+\mu 2^{\ell}} C \\ &\leq r^{n} C \sum_{i=0}^{\mu} K^{i+\ell+1} r^{i 2^{\ell}} \\ &\leq r^{n} C \sum_{i=0}^{\infty}K^{i+\ell+1} r^{i 2^{\ell}} \\ &= r^{n} C \frac{K^{\ell+1}}{1 - K r^{2^{\ell}}} . \end{aligned}$$

Therefore $\{ x_{n} \}$ is Cauchy. □

## Example, part 1

In this section, we give a typical example of a *b*-metric space.

### Lemma 7

*Define a function* *f* *by* (2). *Then the following hold*:

(i) $f(2 n) = f(n) + 1$ *for any* $n \in \mathbb {N}$.
(ii) $f(n+1) \in\{ f(n), f(n)+1 \}$ *for any* $n \in \mathbb {N}$.
(iii) *f* *is nondecreasing*.

### Proof

Obvious. □

### Lemma 8

*Let* $K \in[1,\infty)$ *and put* $X = \mathbb {N}$. *Define a function* *f* *by* (2). *Define a function* *g* *from* $\mathbb {N}\cup\{ 0 \}$ *into* $[0,\infty)$ *by*

5 $$\begin{aligned}& \begin{aligned} & g(0) = 0, \\ & g(n) = \bigl(2 n - 2^{f(n)}\bigr) K^{f(n)} + \bigl(2^{f(n)} - n\bigr) K^{f(n)-1} . \end{aligned} \end{aligned}$$

*Then*

6 $$\begin{aligned} &g(n) = K \bigl( g\bigl([n/2]\bigr) + g\bigl(n-[n/2]\bigr) \bigr) , \end{aligned}$$

7 $$\begin{aligned} &g(n) - g(n-1) \geq g(n-1) - g(n-2) > 0, \end{aligned}$$

8 $$\begin{aligned} &g(n) \leq K \bigl( g(k) + g(n-k) \bigr) \end{aligned}$$

*hold for any* $n,k \in \mathbb {N}$ *with* $2 \leq n$ *and* $k < n$. *Also* *g* *is strictly increasing*.

### Remark

The function *g* is as follows:

$$\begin{aligned} & g(1) = 1 , \quad\quad g(2) = 2 K , \quad\quad g(3) = 2 K^{2} + K , \\ & g(4) = 4 K^{2} , \quad\quad g(5) = 2 K^{3} + 3 K^{2} , \quad\quad g(6) = 4 K^{3} + 2 K^{2} . \end{aligned}$$

### Proof

We first show (6). Fix $n \in \mathbb {N}$. We consider the following two cases:

- $2 n + 1 = 2^{f(2 n + 1)-1} + 1$,
- $2 n + 1 > 2^{f(2 n + 1)-1} + 2$.

We put $a = 2 n + 1$, $b = [(2 n + 1)/2]$ and $c = a - b$. In the first case, noting $f(a) \geq f(3) = 2$, we have

$$ b = \bigl[\bigl(2^{f(2 n + 1)-1} + 1\bigr) / 2\bigr] = 2^{f(2 n + 1)-2} $$

and

$$ c = 2^{f(2 n + 1)-1} + 1 - 2^{f(2 n + 1)-2} = 2^{f(2 n + 1)-2} + 1 . $$

Hence

$$ f(b) = f(2 n + 1)-2 = f(a) - 2 $$

and

$$ f(c) = f(2 n + 1)-1 = f(a) - 1 $$

hold. We have

$$\begin{aligned} g(a) &= \bigl( 2 (2 n+1) - 2^{f(2 n+1)} \bigr) K^{f(a)} + \bigl(2^{f(2 n+1)} - 2 n - 1\bigr) K^{f(a)-1} \\ &= \bigl( 2^{f(2 n+1)} + 2 - 2^{f(2 n+1)} \bigr) K^{f(a)} + \bigl(2^{f(2 n+1)} - 2^{f(2 n+1)-1} - 1\bigr) K^{f(a)-1} \\ &= 2 K^{f(a)} + (a-2) K^{f(a)-1} \\ &= 2 K^{f(c)+1} + b K^{f(b)+1} + (c-2) K^{f(c)} \\ &= K \bigl( \bigl(2 b - 2^{f(b)}\bigr) K^{f(b)} + \bigl(2^{f(b)} - b\bigr) K^{f(b)-1} \\ &\quad{}+ \bigl(2 c - 2^{f(c)}\bigr) K^{f(c)} + \bigl(2^{f(c)} - c\bigr) K^{f(c)-1} \bigr) \\ &= K \bigl( g(b) + g(c) \bigr) . \end{aligned}$$

In the second case, noting that *a* is odd, we have $2^{f(a)-1} > a/2 > 2^{f(a)-2} + 1 $. So

$$ 2^{f(a)-2} < 2^{f(a)-2} + 1 \leq b < a/2 < b+1 = c \leq2^{f(a)-1} $$

holds and hence $f(b) = f(c) = f(a) - 1$ holds. So we have

$$\begin{aligned} g(a) &= \bigl( 2 b+ 2 c - 2^{f(b)} - 2^{f(c)} \bigr) K^{f(a)} + \bigl(2^{f(b)} + 2^{f(c)}- b - c\bigr) K^{f(a)-1} \\ &= \bigl( 2 b - 2^{f(b)} \bigr) K^{f(b)+1} + \bigl( 2^{f(b)} - b \bigr) K^{f(b)} \\ &\quad{}+ \bigl( 2 c - 2^{f(c)} \bigr) K^{f(c)+1} + \bigl( 2^{f(c)} - c\bigr) K^{f(c)} \\ &= K \bigl( g(b) + g(c) \bigr) . \end{aligned}$$

Using Lemma 7, we have

$$\begin{aligned} g(2 n) &= \bigl(4 n - 2^{f(2 n)}\bigr) K^{f(2 n)} + \bigl(2^{f(2 n)} - 2 n\bigr) K^{f(2 n)-1} \\ &= \bigl(4 n - 2^{f(n)+1}\bigr) K^{f(n)+1} + \bigl(2^{f(n)+1} - 2 n\bigr) K^{f(n)} \\ &= 2 K \bigl( \bigl(2 n - 2^{f(n)}\bigr) K^{f(n)} + \bigl(2^{f(n)} - n\bigr) K^{f(n)-1} \bigr) \\ &= K \bigl( g(n) + g(n) \bigr) \\ &= K \bigl( g\bigl([2 n/2]\bigr) + g\bigl(n - [2 n/2]\bigr) \bigr) . \end{aligned}$$

We have shown (6).

We shall show (7). We have

$$ g(2) - g(1) = 2 K - 1 \geq 1 = g(1) - g(0) > 0 . $$

Fix $n \in \mathbb {N}$ with $n \geq2$. In the case where $f(n) = f(n-1)$, we have

$$ g(n) - g(n-1) = 2 K^{f(n)} - K^{f(n)-1} . $$

In the other case, where $f(n) > f(n-1)$, we have $n-1 = 2^{f(n)-1}$ and $f(n-1) = f(n)-1$. So

$$\begin{aligned} g(n) - g(n-1) &= 2 K^{f(n)} + (n-2) K^{f(n)-1} - (n-1) K^{f(n-1)} \\ &= 2 K^{f(n)} - K^{f(n)-1} . \end{aligned}$$

Since *f* is nondecreasing and $K \geq1$ holds, $\{ g(n) - g(n-1) \}$ is also nondecreasing.

Let us prove (8). By (7), there exists a convex function from $[0,\infty)$ into $\mathbb {R}$ whose restriction to $\mathbb {N}$ is *g*. So we have

$$\begin{aligned} g(n) &= K \bigl( g\bigl([n/2]\bigr) + g\bigl(n-[n/2]\bigr) \bigr) \\ &\leq K \bigl( g\bigl([n/2]-1\bigr) + g\bigl(n-[n/2]+1\bigr) \bigr) \\ &\leq K \bigl( g\bigl([n/2]-2\bigr) + g\bigl(n-[n/2]+2\bigr) \bigr) \\ &\leq\cdots \leq K \bigl( g(1) + g(n-1) \bigr) . \end{aligned}$$

We have shown (8).

By (7), *g* is strictly increasing. □

The following example is a typical example of a *b*-metric space. Indeed, we use this example in order to make Example 11.

### Example 9

Let $K \in[1,\infty)$ and put $X = \mathbb {N}$. Define functions *f* and *g* by (2) and (5), respectively. Define a function *d* from $X \times X$ into $[0,\infty)$ by

$$ d(x,y) = g\bigl(\vert x-y\vert \bigr) . $$

Then $(X,d)$ is a *b*-metric space.

### Proof

(b1) and (b2) obviously hold. In order to prove (b3), we note

$$ d(\ell,m) \leq d(\ell,n) \quad\text{and}\quad d(m,n) \leq d(\ell,n) $$

for any $\ell,m,n \in \mathbb {N}$ with $\ell< m < n$ because *g* is strictly increasing. Fix $\ell, m, n \in \mathbb {N}$ with $\ell< n$, $\ell\neq m$ and $m \neq n$. We consider the following two cases:

- $m < \ell$ or $n < m$,
- $\ell< m < n$.

In the case of (a), without loss of generality, we may assume $n < m$. Then we have

$$ d(\ell, n) \leq d(\ell, m) \leq d(\ell, m) + d(m, n) \leq K d(\ell, m) + K d(m, n) . $$

In the case of (b), we have by (8)

$$ d(\ell,n) \leq K d(\ell,m) + K d(m,n) . $$

We have shown (b3). □

## Examples, part 2

We have proved that (4) implies the Cauchyness on sequences. In this section, we give examples of that $\sum_{n=1}^{\infty}d(x_{n}, x_{n+1}) < \infty$ does not imply the Cauchyness on sequences. The author thinks that such a property is one of characteristics of a *b*-metric space.

### Example 10

Let $K \in[1,\infty)$ and let $\{ \alpha_{n} \}$ be a sequence in $(0,\infty)$. Let *X* be a subset of $[1,\infty)$ satisfying $\mathbb {N}\subset X$ and $\# (X \cap[1,a]) < \infty$ for any $a \in [1,\infty)$. Define a strictly increasing function *χ* from $\mathbb {N}$ into *X* satisfying $\chi(\mathbb {N}) = X$. Define a sequence $\{ \nu_{j} \}$ in $\mathbb {N}$ satisfying $\chi(\nu_{j}) = j$ for any $j \in \mathbb {N}$. Define functions *f* and *g* by (2) and (5), respectively. Define a function *d* from $X \times X$ into $[0,\infty)$ by

$$ d \bigl( \chi(m), \chi(n) \bigr) = \textstyle\begin{cases} 0 \hspace{59pt}\text{if } m = n, \\ g(n-m) \alpha_{k} \hspace{21pt}\text{if } \nu_{k} \leq m < n \leq\nu_{k+1} \text{ for some } k \in \mathbb {N},\\ d ( \chi(m), j+1 ) + \sum_{i=j+1}^{k-1} d(i, i+1) + d ( k, \chi(n) ) \\ \hspace{65pt} \text{if } \nu_{j} \leq m < \nu_{j+1} \leq\nu_{k} < n \leq\nu_{k+1} \text{ for some } j, k \in \mathbb {N},\\ d ( \chi(n), \chi(m) ) \quad \text{if } m > n, \end{cases} $$

for all $m, n \in \mathbb {N}$. Then $(X,d)$ is a *b*-metric space.

### Proof

It is obvious that (b1) and (b2) hold. Let us prove (b3). Define a function *e* from $\mathbb {N}\times \mathbb {N}$ into $[0,\infty)$ by

$$ e(m,n) = d \bigl( \chi(m), \chi(n) \bigr) . $$

We note

$$ e(\ell,m) \leq e(\ell,n) \quad\text{and}\quad e(m,n) \leq e(\ell,n) $$

for any $\ell, m, n \in \mathbb {N}$ with $\ell< m < n$. Fix $\ell, m, n \in \mathbb {N}$ with $\ell< n$, $\ell\neq m$ and $m \neq n$. We consider the following three cases:

- $m < \ell$ or $n < m$,
- $\nu_{k} \leq\ell< m < n \leq\nu_{k+1}$ for some $k \in \mathbb {N}$,
- $\ell< m < n$ and $\nu_{j} \leq\ell< \nu_{j+1} \leq\nu_{k} < n \leq\nu_{k+1} $ for some $j, k \in \mathbb {N}$.

In the cases of (a) and (b), we can prove (b3) as in the proof of Example 9. In the case of (c), we further consider the following two cases:

1. $m \leq\nu_{j+1}$ or $\nu_{k} \leq m$,
2. $\nu_{j+1} < m < \nu_{k}$.

In the case of (c-1), without loss of generality, we may assume $m \leq\nu_{j+1}$. We have

$$\begin{aligned} e(\ell,n) &= e(\ell,\nu_{j+1}) + \sum_{i=j+1}^{k-1} e(\nu_{i}, \nu_{i+1}) + e(\nu_{k}, n) \\ &\leq K e(\ell,m) + K e(m, \nu_{j+1}) + \sum _{i=j+1}^{k-1} e(\nu_{i}, \nu_{i+1}) + e(\nu_{k}, n) \\ &\leq K e(\ell,m) + K e(m, \nu_{j+1}) + K \sum _{i=j+1}^{k-1} e(\nu_{i}, \nu_{i+1}) + K e(\nu_{k}, n) \\ &= K \bigl( e(\ell,m) + e(m,n) \bigr) . \end{aligned}$$

In the case of (c-2), there exists $p \in \mathbb {N}$ satisfying

$$ \nu_{j+1} \leq\nu_{p} \leq m < \nu_{p+1} \leq\nu _{k} . $$

We have

$$\begin{aligned} e(\ell, n) &= e(\ell,\nu_{j+1}) + \sum_{i=j+1}^{p-1} e(\nu_{i}, \nu_{i+1}) \\ &\quad{} + e(\nu_{p}, \nu_{p+1}) + \sum _{i=p+1}^{k-1} e(\nu_{i}, \nu_{i+1}) + e(\nu_{k}, n) \\ &\leq e(\ell,\nu_{j+1}) + \sum_{i=j+1}^{p-1} e(\nu_{i}, \nu_{i+1}) \\ &\quad{} + K e(\nu_{p}, m) + K e(m, \nu_{p+1}) + \sum _{i=p+1}^{k-1} e(\nu_{i}, \nu_{i+1}) + e(\nu_{k}, n) \\ &\leq K e(\ell,\nu_{j+1}) + K \sum_{i=j+1}^{p-1} e(\nu_{i}, \nu_{i+1}) \\ &\quad{} + K e(\nu_{p}, m) + K e(m, \nu_{p+1}) + K \sum _{i=p+1}^{k-1} e(\nu_{i}, \nu_{i+1}) + K e(\nu_{k}, n) \\ &= K \bigl( e(\ell,m) + e(m,n) \bigr) . \end{aligned}$$

We have shown (b3) in all cases. □

### Example 11

Let $K \in(1,\infty)$ and define a sequence $\{ \alpha_{n} \}$ in $(0,\infty)$ by $\alpha_{n} = 2^{-n} K^{-n}$. Let *X* be a subset of $[1,\infty)$ satisfying $\mathbb {N}\subset X$ and $\# ( X \cap[n,n+1) ) = 2^{n}$ for any $n \in \mathbb {N}$. Let *χ*, $\{ \nu_{j} \}$, *f*, *g* and *d* be as in Example 10. Then the following hold:

(i) $(X,d)$ is a *b*-metric space.
(ii) $\sum_{n=1}^{\infty}d ( \chi(n), \chi(n+1) ) < \infty$ holds.
(iii) $\{ \chi(n) \}$ is not Cauchy.
(iv) *X* is complete.

### Proof

We have proved (i) in Example 10. We have

$$\begin{aligned} \sum_{n=1}^{\infty}d \bigl( \chi(n), \chi(n+1) \bigr) &= \sum_{k=1}^{\infty}\sum _{n=\nu_{k}}^{\nu_{k+1}-1} d \bigl( \chi (n), \chi(n+1) \bigr) \\ &= \sum_{k=1}^{\infty}\sum _{n=\nu_{k}}^{\nu_{k+1}-1} \alpha_{k} = \sum _{k=1}^{\infty}2^{k} \alpha_{k} = \sum _{k=1}^{\infty}K^{-k} < \infty. \end{aligned}$$

We have

$$ d(n, n+1) = g\bigl(2^{n}\bigr) \alpha_{n} = 2^{n} K^{n} \alpha_{n} = 1 $$

for any $n \in \mathbb {N}$. Since the sequence $\{ n \}$ in *X* is a subsequence of $\{ \chi(n) \}$, $\{ \chi(n) \}$ is not Cauchy.

Let us prove (iv). We note $d(n,m) = \vert n - m \vert $ for any $m, n \in \mathbb {N}$. So $\lim_{m} d ( \chi(n), \chi(m) ) = \infty$ holds for any $n \in \mathbb {N}$. Let $\{ x_{n} \}$ be a Cauchy sequence in *X*. Then $\{ x_{n} \}$ is bounded. So, there exists $z \in X$ satisfying $x_{n} = z$ for sufficiently large $n \in \mathbb {N}$. Thus, $\{ x_{n} \}$ converges to *z*. □

## Fixed point theorems

Czerwik proved the following fixed point theorem. See page 126 of [8]. Compare Theorem 12 with Theorem 1 in [9] and Theorem 2.1 in [10].

### Theorem 12

([1])

*Let* $(X,d)$ *be a complete* *b*-*metric space and let* *T* *be a mapping from* *X* *into* $\operatorname {CB}(X)$. *Assume that there exists* $r \in[0,1/K)$ *satisfying*

9 $$ H(Tx,Ty) \leq r d(x, y) $$

*for all* $x, y \in X$. *Then there exists* $z \in X$ *satisfying* $z \in Tz$.

Using Lemma 6, we improve Theorem 12. We begin with a generalization of Theorem 3 in [11].

### Theorem 13

*Let* $(X, d)$ *be a complete* *b*-*metric space and let* *T* *be a mapping from* *X* *into* $\operatorname {CB}(X)$. *Assume there exists* $\varepsilon> 0$ *satisfying the following*:

(i) *There exists* $r \in[0, 1)$ *such that* (9) *holds for all* $x, y \in X$ *with* $d(x, y) < \varepsilon$.
(ii) *There exists* $x \in X$ *such that* $d(x, Tx) < \varepsilon$.

*Then there exists* $z \in X$ *satisfying* $z \in Tz$.

### Proof

Put $q := (1+r)/2 \in(0,1)$. Then we have the following:

- For $x, y \in X$ and $u \in Tx$ with $d(x,y) < \varepsilon$, there exists $v \in Ty$ satisfying $d(u, v) \leq q d(x, y) $.

In particular, putting $u = y$, we obtain the following:

- For $x \in X$ and $y \in Tx$ with $d(x,y) < \varepsilon$, there exists $v \in Ty$ satisfying $d(y, v) \leq q d(x, y) $.

Therefore we can choose a sequence $\{ u_{n} \}$ in *X* satisfying

$$\begin{aligned} & d(u_{1}, T u_{1}) \leq d(u_{1}, u_{2}) < \varepsilon, \\ & u_{n+1} \in T u_{n} , \\ & d(u_{n+1}, u_{n+2}) \leq q d(u_{n}, u_{n+1}) \end{aligned}$$

for $n \in \mathbb {N}$. By Lemma 6, $\{ u_{n} \}$ is Cauchy. Since *X* is complete, $\{ u_{n} \}$ converges to some point $z \in X$. Since

$$\begin{aligned} d(z, Tz) &\leq\lim_{n \to\infty} K \bigl( d(z, u_{n+1}) + d(u_{n+1}, Tz) \bigr) \\ &= K \lim_{n \to\infty} d(u_{n+1}, Tz) \leq K \lim _{n \to\infty} H(T u_{n}, Tz) \\ &\leq K q \lim_{n \to\infty} d(u_{n}, z) = 0 \end{aligned}$$

holds and *Tz* is closed, we obtain $z \in Tz$. □

As a direct consequence, we obtain a generalization of Nadler’s fixed point theorem [12].

### Corollary 14

*Let* $(X, d)$ *be a complete* *b*-*metric space and let* *T* *be a mapping from* *X* *into* $\operatorname {CB}(X)$. *Assume that there exists* $r \in[0, 1)$ *such that* (9) *holds for all* $x, y \in X$. *Then there exists* $z \in X$ *satisfying* $z \in Tz$.

Using Theorem 13, we can prove a generalization of Mizoguchi and Takahashi’s fixed point theorem [13]. See also [11, 14–16] and others.

### Corollary 15

*Let* $(X, d)$ *be a complete* *b*-*metric space and let* *T* *be a mapping from* *X* *into* $\operatorname {CB}(X)$. *Assume that there exists a function* *α* *from* $[0, \infty)$ *into* $[0, 1)$ *satisfying*

$$ H(Tx, Ty) \leq\alpha \bigl( d(x, y) \bigr) d(x, y) $$

*for all* $x, y \in X$ *and*

$$ \limsup_{s \to t + 0} \alpha(s) < 1 $$

*for all* $t \in[0, \infty)$. *Then there exists* $z \in X$ *satisfying* $z \in Tz$.

### Proof

Since $\limsup_{s \to+0} \alpha(s) < 1$, we can choose $\varepsilon> 0$ and $r \in[0,1)$ satisfying $\alpha(t) \leq r$ for any $t \in[0,\varepsilon)$. Thus (i) of Theorem 13 holds. So we only have to prove (ii) of Theorem 13. Define a function *β* from $[0, \infty)$ into $(0, 1)$ by $\beta(t) = ( \alpha(t) + 1 ) / 2$ for $t \in[0, \infty)$. As in the proof of Theorem 13, we can choose a sequence $\{ u_{n} \}$ in *X* satisfying

$$ u_{n+1} \in T u_{n} \quad\text{and}\quad d(u_{n+1}, u_{n+2}) \leq\beta \bigl( d(u_{n}, u_{n+1}) \bigr) d(u_{n}, u_{n+1}) $$

for $n \in \mathbb {N}$. Since $\beta(t) < 1$ for any $t \in[0, \infty)$, $\{ d(u_{n}, u_{n+1}) \}$ is a nonincreasing sequence in $[0,\infty)$. So $\{ d(u_{n}, u_{n+1}) \}$ converges to some $\tau\in[0,\infty)$. Since $\limsup_{s \to\tau+ 0} \beta(s) < 1$ and $\beta(\tau) < 1$, there exist $q \in[0, 1)$ and $\delta> 0$ satisfying $\beta(s) \leq q $ for all $s \in[\tau, \tau+\delta]$. Choose $\nu\in \mathbb {N}$ satisfying $d(u_{\nu}, u_{\nu+1}) \leq\tau+ \delta$. Then, for any $n \in \mathbb {N}$ with $n \geq\nu$, we have

$$ d(u_{n+1}, u_{n+2}) \leq\beta \bigl( d(u_{n}, u_{n+1}) \bigr) d(u_{n}, u_{n+1}) \leq q d(u_{n}, u_{n+1}) , $$

which implies $\lim_{n} d(u_{n}, u_{n+1}) = 0$. Therefore $d(u_{n}, u_{n+1}) \leq d(u_{n}, T u_{n}) < \varepsilon$ holds for sufficiently large $n \in \mathbb {N}$. We have shown (ii) of Theorem 13. □
